# LncRNA MAFG-AS1 is involved in human cancer progression

**DOI:** 10.1186/s40001-023-01486-9

**Published:** 2023-11-08

**Authors:** Penghui Li, Xiao Ma, Xinyu Gu

**Affiliations:** 1 Department of Oncology, The First Affiliated Hospital, College of Clinical Medicine, Henan University of Science and Technology, Luoyang, 471000 Henan China; 2 Zhejiang University School of Medicine, Hangzhou, 310000 Zhejiang China

**Keywords:** lncRNA, MAFG-AS1, Cancer, Expression, Function

## Abstract

Long noncoding RNAs (lncRNAs) refer to a type of non-protein-coding transcript of more than 200 nucleotides. LncRNAs play fundamental roles in disease development and progression, and lncRNAs are dysregulated in many pathophysiological processes. Thus, lncRNAs may have potential value in clinical applications. The lncRNA, MAF BZIP Transcription Factor G (MAFG)-AS1, is dysregulated in several cancer, including breast cancer, lung cancer, liver cancer, bladder cancer, colorectal cancer, gastric cancer, esophagus cancer, prostate cancer, pancreatic cancer, ovarian cancer, and glioma. Altered MAFG-AS1 levels are also associated with diverse clinical characteristics and patient outcomes. Mechanistically, MAFG-AS1 mediates a variety of cellular processes via the regulation of target gene expression. Therefore, the diagnostic, prognostic, and therapeutic aspects of MAFG-AS1 have been widely explored. In this review, we discuss the expression, major roles, and molecular mechanisms of MAFG-AS1, the relationship between MAFG-AS1 and clinical features of diseases, and the clinical applications of MAFG-AS1.

## Introduction

Cancer represents a group of heterogeneous diseases, which involve uncontrolled growth of mutated cells, invasion of adjacent organs, and distant metastasis [1–4]. The application of discoveries and innovations in molecular cancer therapies has significantly improved patient prognoses [5–9]. However, the high incidence and mortality of cancer are still a public health concern [10–13]. New molecular mechanisms and strategies are still needed to improve therapeutic responses and clinical outcomes [14–18].

Along with advances in high-throughput sequencing technology, an increasing population of noncoding RNAs (ncRNAs) has been discovered [19–22], including long noncoding RNAs (lncRNAs) [23–28]. LncRNAs are transcripts of at least 200 nucleotides that do not have protein-coding capability [23, 29–31]. LncRNA dysregulation is involved in diverse human diseases, including neurological diseases, cardiovascular diseases, and cancers [32–34], and diverse cellular processes [35–38], including cell proliferation, differentiation, apoptosis, and migration. In addition, lncRNAs regulate the expression of protein-coding genes and, thus, foster the progression of diseases or tumors. Given these properties, a large proportion of lncRNAs are important in disease diagnosis, prognosis, and therapeutic targets [39–42].

The lncRNA MAF BZIP Transcription Factor G (MAFG)-AS1, located on human chromosome 17q25.3, was recently identified as an oncogenic lncRNA with a transcript size of 1895 bp. MAFG-AS1 expression is aberrant in diverse diseases, including breast cancer [43–49], lung cancer [50–52], liver cancer [53–58], bladder cancer [59–63], colorectal cancer [64–66], gastric cancer [67, 68], esophagus cancer [69], prostate cancer [70], pancreatic cancer [71], ovarian cancer [72], glioma [73], periodontitis [74], and coronary artery disease [75]. MAFG-AS1 levels are also strongly associated with clinicopathological characteristics and patient outcomes, such as tumor size, clinical stage, distant metastasis, overall survival (OS), and disease‐free survival (DFS). Experimental studies demonstrate the involvement of MAFG-AS1 in disease development via a series of biological processes, such as cell proliferation, invasion, glycolysis, metastasis, and drug sensitivity. MAFG-AS1 affects cancer progression by regulating target gene expression.

In the present review, we discuss the expression, related clinical features, and biological functions of MAFG-AS1 in diverse cancers. In addition, we discuss the underlying mechanisms and clinical applications of MAFG-AS1.

## Characteristics of MAFG-AS1 in human cancers

**Fig. 1:**
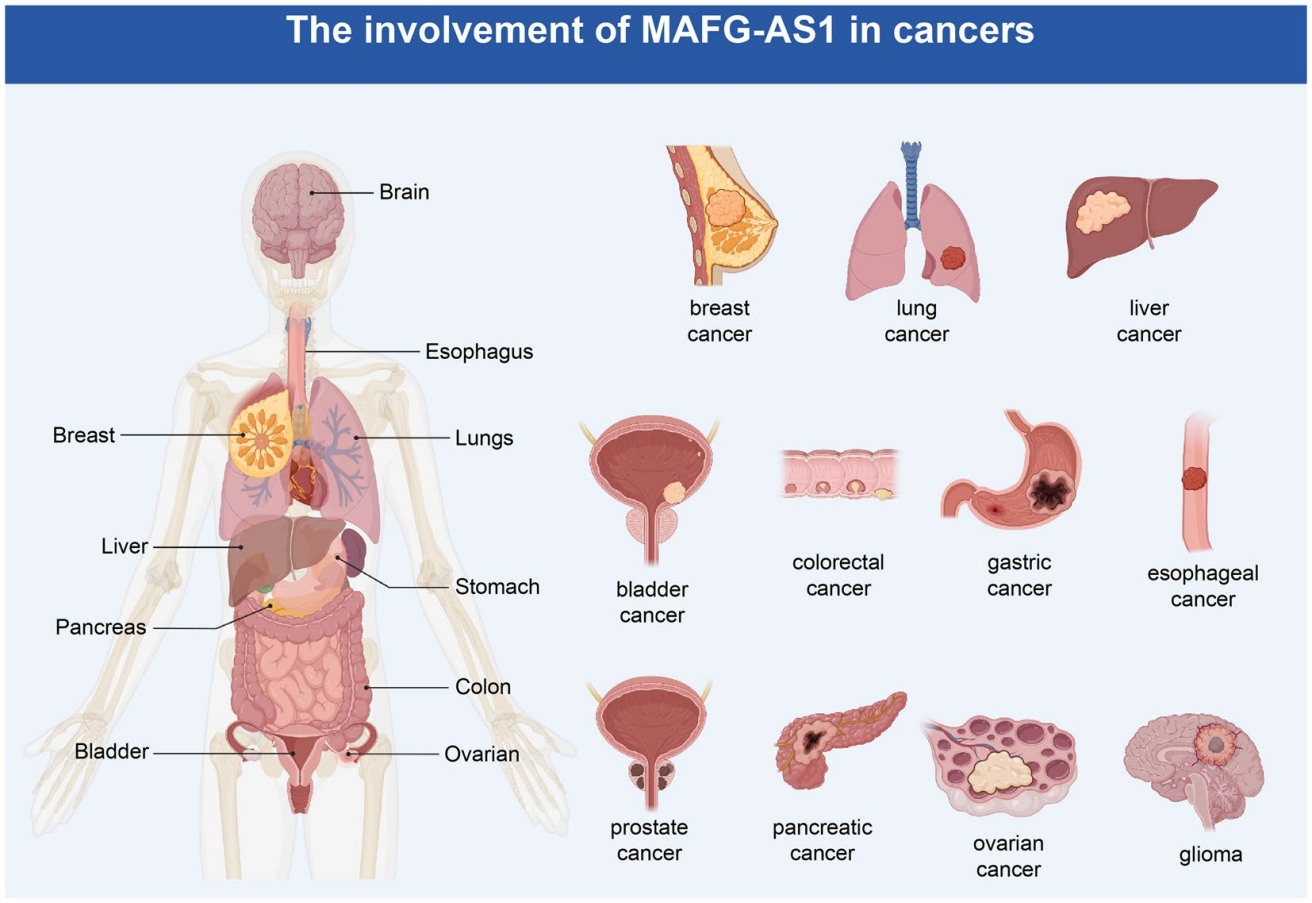
The involvement of MAFG-AS1 in human diseases. MAFG-AS1 is dysregulated in breast cancer, lung cancer, liver cancer, bladder cancer, colorectal cancer, gastric cancer, esophagus cancer, prostate cancer, pancreatic cancer, ovarian cancer, and glioma

MAFG-AS1 is dysregulated in diverse diseases, including breast cancer, lung cancer, liver cancer, bladder cancer, colorectal cancer, gastric cancer, esophagus cancer, prostate cancer, pancreatic cancer, ovarian cancer, and glioma (Fig. 1). High MAFG-AS1 expression correlates with unfavorable clinical features and prognosis, including lymph node metastasis, histological grade, clinical stage, distant metastasis, OS, and DFS (Table 1). Importantly, MAFG-AS1 often functions as a sponge to interfere with microRNA regulation of gene expression, which affects many biological processes, including cell proliferation, invasion, glycolysis, metastasis, and drug sensitivity (Table 2). In this section, we include a comprehensive description of the relationship between MAFG-AS1 expression and clinical features of diverse cancers.

**Table 1:** The expression and clinical characteristics of MAFG-AS1 in cancers

Disease type	Expression	Clinical characteristics	Refs.
Breast cancer	Upregulated	Tumor size, and ki-67	[43–49]
Lung cancer	Upregulated	OS	[50–52]
Liver cancer	Upregulated	OS	[53–58]
Bladder cancer	Upregulated	DFS, OS, clinical stages, and TMN stages	[59–63]
Colorectal cancer	Upregulated	Depth of invasion, TNM stage, OS, and DFS	[64–66]
Gastric cancer	Upregulated	Clinical stage, depth of invasion, lymph node metastasis, distant metastasis, and OS	[67, 68]
Esophageal cancer	Upregulated	OS	[69]
Prostate cancer	Upregulated	–	[70]
Pancreatic cancer	Upregulated	–	[71]
Ovarian cancer	Upregulated	Tumor stage, size, lymph node metastasis, and OS	[72]
Glioma	Upregulated	–	[73]

**Table 2:** The roles and mechanisms of MAFG-AS1 in cancers

Disease type	Cell lines	Functions	Related mechanisms	Refs.
Breast cancer	MCF7, MCF10, SUM149, HCC1937, BT474, Hs578T, SK-BR-3, MDA-MB-468, MDA-MB-231, and T47D	Cell proliferation, invasion, metastasis, apoptosis, and autophagy	miR-3612, FKBP4, miR-574-5p, SOD2, miR-150-5p, MYB, miR-339-5p, CDK2, miR-339-5p, MMP15, miR-3196, TFAP2A, JAK2, STAT3, and STC2	[43–49]
Lung cancer	H1373, H1975, H1650, HCC827, A549, PC-9, and Calu-3	Cell proliferation, migration, and invasion	miR-339-5p, MMP15, miR-744-5p, MAFG, miR-3196, and SOX12	[50–52]
Liver cancer	Huh7, HepG2, LM3, HCCLM3, Hep3B, and MHCC97-H	Cell proliferation, migration, invasion, and drug resistance	miR-3196, STRN4, E2F1, MAFG, miR-3196, OTX1, and miR-6852	[53–58]
Bladder cancer	HT01197, 5637, BIU87, EJ, RT4, J82, T24, HT-1376, UMUC3, and SVHUC1	Cell proliferation, migration, and invasion	miR-125b-5p, SphK1, HuR, PTBP1, miR-143-3p, COX-2, miR-143-3p, and SERPINE1	[59–63]
Colorectal cancer	HCT116, HT29, SW1116, SW480, and LoVo	Cell migration, proliferation, invasion, and glycolysis	miR-147b, NDUFA4, miRNA-149-3p, and HOXB8	[64–66]
Gastric cancer	MKN-45, AGS, and SGC7901	Cell proliferation, migration, and invasion	miR-505, and PLK1	[67, 68]
Esophageal cancer	EC9706, EC109, KYSE30, and KYSE150	Cell proliferation, migration, invasion, and aerobic glycolysis	miR-765, and PDX1	[69]
Prostate cancer	DU145, and PC-3	Cell proliferation and invasion	miR-3196, and NFIX	[70]
Pancreatic cancer	Capan 1, CFPAC-1, SW1990, and PANC-1	Cell proliferation and migration	NFKB1, and IGF1	[71]
Ovarian cancer	A2780, Caov-3, RMG-I, Caov-4, and CoC1	Cell invasion and migration	–	[72]
Glioma	U87, and U-118	Cell proliferation	miR-34a	[73]

### Breast cancer

MAFG-AS1 overexpression in breast cancer tissue and cells (MCF7, MCF10, SUM149, HCC1937, BT474, Hs578T, SK-BR-3, MDA-MB-468, MDA-MB-231, and T47D) [43–49] revealed that MAFG-AS1 levels positively correlate with tumor size and ki-67 index [48]. MAFG-AS1 participates in cancer progression via enhanced cell proliferation, invasion, and metastasis and suppression of cell apoptosis and autophagy. Studies using xenograft models confirm the pro‐oncogenic roles of MAFG-AS1 in tumor growth and lung metastasis [44, 46–48].

### Lung cancer

MAFG-AS1 is also upregulated in lung cancer tissues and H1373, H1975, H1650, HCC827, A549, PC-9, and Calu-3 cells [50–52]. High MAFG-AS1 levels are associated with poor prognosis in patients with lung cancer. MAFG-AS1 increases cell proliferation, migration, invasion, and tumor‐forming and metastasis abilities to advance lung cancer [50–52].

### Liver cancer

MAFG-AS1 upregulation in liver cancer tissues and Huh7, HepG2, LM3, HCCLM3, Hep3B, and MHCC97-H cells is associated with shorter OS [53–58]. MAFG-AS1 exerts its pro-cancer roles via increased cell proliferation, migration, invasion, drug resistance, and tumor angiogenesis [54–56, 58].

### Bladder cancer

MAFG-AS1 is upregulated in bladder cancer cells (HT01197, 5637, BIU87, EJ, RT4, J82, T24, HT-1376, UMUC3, and SVHUC1) and tissues [59–63]. MAFG-AS1 upregulation correlates with aggressive prognosis, shorter DFS and OS, and advanced clinical and TMN stages. Both in vivo and in vitro experimental studies demonstrate that the upregulation of MAFG-AS1 increases proliferation, migration, and invasion, which contribute to the development of bladder cancer.

### Colorectal cancer

MAFG-AS1 levels are significantly increased in colorectal cancer tissues and HCT116, HT29, SW1116, SW480, and LoVo cells [64–66]. High MAFG-AS1 expression is closely related to invasion depth, advanced TNM stage, and shorter OS and DFS [65, 66]. MAFG-AS1 also facilitates cell migration, proliferation, invasion, glycolysis, and tumor growth to promote colorectal cancer [64, 66].

### Gastric cancer

MAFG-AS1 is overexpressed in gastric cancer MKN-45, AGS, and SGC7901 cells and tissues. MAFG-AS1 upregulation is associated with deteriorative clinical stage, depth of invasion, lymph node metastasis, distant metastasis, and unfavorable OS [67, 68]. MAFG-AS1 also plays a pro-cancer role in gastric cancer through the promotion of cell proliferation, migration, and invasion.

### Other cancers

MAFG-AS1 is upregulated in esophageal cancer tissues and cells (EC9706, EC109, KYSE30, and KYSE150) and is associated with shorter OS. MAFG-AS1 enhances cell proliferation, migration, invasion, and aerobic glycolysis and, thus, exerts cancer-promoting effects in esophageal cancer [69]. In prostate cancer, MAFG-AS1 is overexpressed in tissues and DU145 and PC-3 cells and participates in cell proliferation and invasion [70]. MAFG-AS1 levels are elevated in pancreatic cancer tissues and Capan 1, CFPAC-1, SW1990, and PANC-1 cells and strengthen cancer development via enhanced cell proliferation and migration [71]. Similarly, increased MAFG-AS1 in ovarian cancer A2780, Caov-3, RMG-I, Caov-4, and CoC1 cells tightly correlates with aggressive tumor stage, size, lymph node metastasis, and poor outcomes. MAFG-AS1 contributes to ovarian tumor progression via enhanced invasion and migration [72]. MAFG-AS1 is upregulated in glioma tissues, and U87 and U-118 cells and is pro-proliferative [73].

## The pro-oncogenic mechanisms of MAFG-AS1 in human cancers

MAFG-AS1 is involved in the development of various cancers and governs numerous biological processes through diverse mechanisms, including cell proliferation, migration, invasion, apoptosis, autophagy, drug resistance, and glycolysis (Table 2). This section briefly introduces the mechanisms for MAFG-AS1 effects in human cancers.

**Fig. 2:**
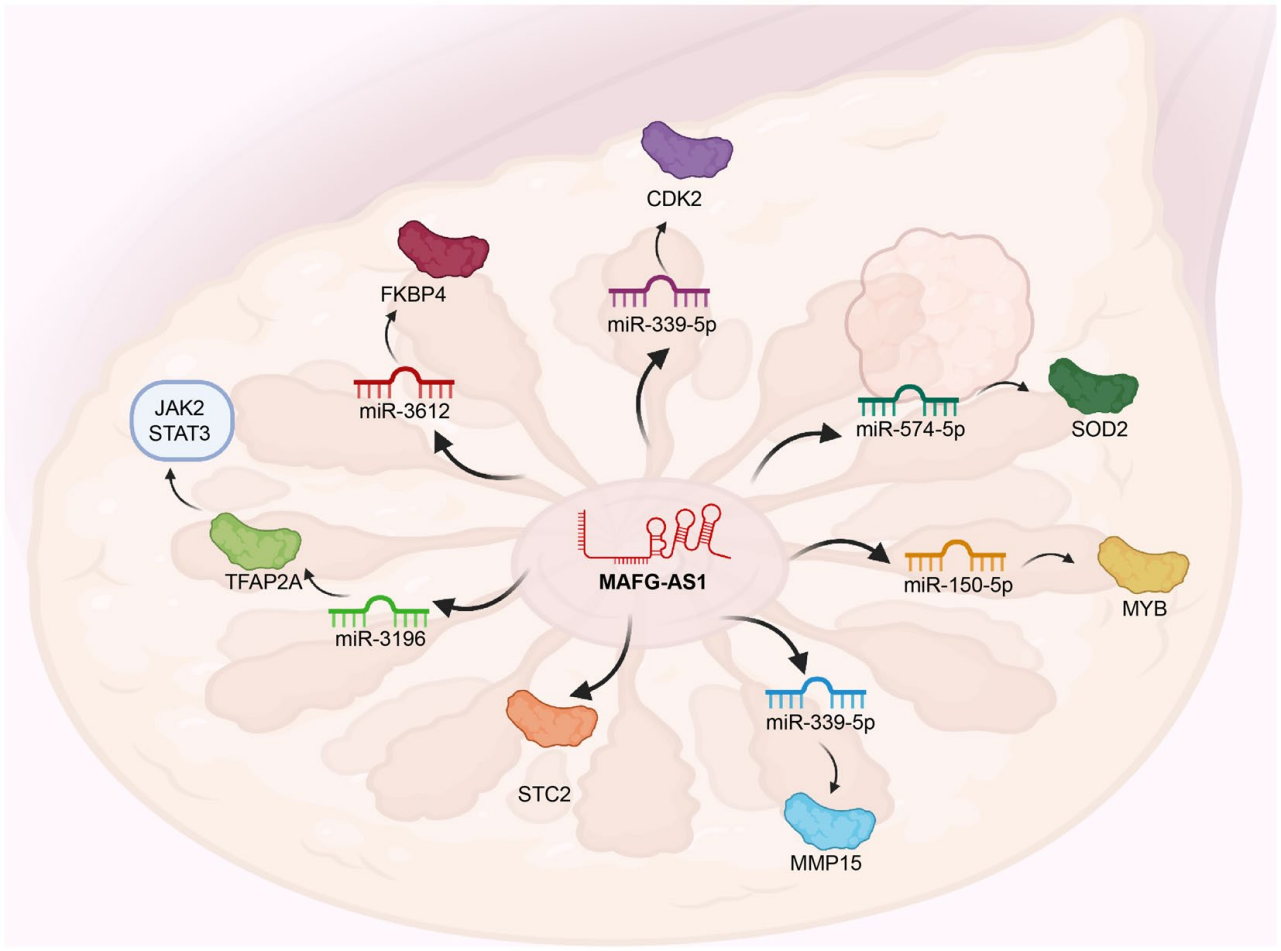
The regulatory mechanisms of MAFG-AS1 in breast cancer progression. MAFG-AS1 plays pro-oncogenic roles in breast cancer through the miR-3612/FKBP4, miR-574-5p/SOD2, miR-150-5p/MYB, miR-339-5p/CDK2, miR-339-5p/MMP15, and miR-3196/TFAP2A/JAK2/STAT3 signaling pathways and upregulates STC2 expression

The impaired regulation of cell proliferation, via defective regulatory pathways, mutations in critical genes, and environmental factors [76–80], contributes to tumor formation. Excessively increased migratory and invasive capacities of cancer cells promote cancer progression and higher mortality rates [81–85]. Moreover, energy metabolism also affects the pathogenesis of cancer [3, 86, 87]. Therefore, understanding the molecular mechanisms that govern cell processes is crucial for subsequent cancer management [88–91]. MAFG-AS1 affects cell proliferation and migration through diverse mechanisms. For example, MAFG-AS1 enhances cell proliferation, invasion, metastasis, and glycolysis to facilitate breast cancer development through multiple mechanisms. MAFG-AS1 plays pro-oncogenic roles through the miR-3612/FKBP4, miR-574-5p/SOD2, miR‑150‑5p/MYB, miR-339-5p/CDK2, miR-339-5p/MMP15, and miR-3196/TFAP2A/JAK2/STAT3 signaling pathways [43, 45–49] (Fig. 2). MAFG-AS1 also stabilizes STC2 expression to promote breast cancer [44]. MAFG-AS1 increases cell proliferation, migration, and invasion of lung cancer cells through the miR-339-5p/MMP15, miR-744-5p/MAFG, and miR-3196/SOX12 axes [50–52]. In liver cancer, MAFG-AS1 sponges miR-3196 to increase STRN4 expression, interacts with E2F1 to enhance MAFG levels, combines with miR-3196 to elevate OTX1 transcription, or decreases miR-6852 to increase cell proliferation, migration, and invasion [54–56, 58]. MAFG-AS1 promotes the proliferation, migration, and invasion of bladder cancer through miR-125b-5p/SphK1, HuR/PTBP1, miR-143-3p/COX-2, and miR-143-3p/SERPINE1 pathways [59, 60, 62, 63]. MAFG-AS1 may also contribute to colorectal cancer cell migration, proliferation, invasion, and glycolysis [64, 66] by binding to miR-147b to activate NDUFA4 or absorbing miRNA-149-3p to increase HOXB8 expression. In addition, MAFG-AS1 upregulates PLK1 by sponging miR-505 to increase gastric cancer cell proliferation migration, and invasion [67]. In esophageal cancer, MAFG-AS1 increases cell proliferation, migration, invasion, and aerobic glycolysis through interactions with miR-765 and the subsequent upregulation of PDX1 [69]. MAFG-AS1 also sponges miR-3196 to increase NFIX expression and enhance pancreatic cancer cell proliferation and migration [71]. In ovarian cancer, MAFG-AS1 upregulates IGF1 expression by interacting with NFKB1 to facilitate cell invasion and migration [72]. MAFG-AS1 elevates the proliferation of gliomas by decreasing the expression of mature miR-34a [73].

## Clinical applications of MAFG-AS1 in human cancers

Despite the continuous strides in disease prevention and treatment, the global burden of cancer remains heavy [92–95]. In this context, new potent and safe molecules are needed to develop combination therapy strategies.

As the roles of MAFG-AS1 in diverse cancers are revealed, its clinical value has received increased attention. Multiple studies show the overexpression of MAFG-AS1 in tissues and cells and its pro-oncogenic roles in many cancers. MAFG-AS1 overexpression helps distinguish between cancerous and normal tissues and improves early-stage cancer diagnosis. Given the close association between MAFG-AS1 and diverse clinical features, MAFG-AS1 is a powerful prognostic tool for cancers. Kaplan–Meier survival curves demonstrate that high MAFG-AS1 levels correlate with patient’s poor prognoses (such as overall survival and progression free survival) in diverse cancers, including breast, lung, liver, bladder, colorectal, gastric, and esophageal cancers [52, 53, 57, 59, 60, 63, 65, 67–69]. Univariate Cox regression analyses in liver and gastric cancer patients also confirm the significant association of MAFG-AS1 with unfavorable OS [53, 57, 68]. Multivariate analyses revealed that MAFG‐AS1 is an independent prognostic biomarker in bladder, colorectal, and gastric cancers [60, 65, 68]. The detection of MAFG‑AS1 levels in cancer tissues and cells may improve the diagnosis and prognosis of several cancers and guide therapeutic approaches. In addition, recent studies suggest that MAFG‑AS1 is involved in important biological processes through diverse molecular mechanisms, especially the regulation of downstream molecules. MAFG‑AS1 knockdown slows cancer progression and is a potential novel therapy. MAFG‑AS1 enhances cancer cell resistance to tamoxifen, which is a target for the treatment of breast cancer [48]. Accordingly, MAFG‑AS1 has great potential in clinical application in terms of cancer diagnosis, prognosis, and therapy. Molecular therapy holds great potential in the field of oncology, albeit with certain challenges [96].

## Conclusions

As a novel tumor-related lncRNA, dysregulation of MAFG‑AS1 contributes to multiple human cancers, including breast cancer, lung cancer, liver cancer, bladder cancer, colorectal cancer, gastric cancer, esophagus cancer, prostate cancer, pancreatic cancer, ovarian cancer, and glioma. Elevated MAFG‑AS1 expression is closely associated with diverse undesirable clinical characteristics and poor outcomes. Multiple experimental studies also revealed that MAFG‑AS1 acts on a variety of targets to mediate crucial biological processes, including cell migration, invasion, proliferation, energy metabolism, and drug resistance. Considering its attractive features in diverse cancers, MAFG‑AS1 possesses wide prospects for clinical applications, including diagnosis, prognosis, and treatment.

## Data Availability

Not applicable.
